# Comparing the effects of vitamin E tocotrienol-rich fraction supplementation and α-tocopherol supplementation on gene expression in healthy older adults

**DOI:** 10.6061/clinics/2019/e688

**Published:** 2019-02-27

**Authors:** Siti Madiani Abdul Ghani, Jo Aan Goon, Nor Helwa Ezzah Nor Azman, Siti Nor Asyikin Zakaria, Zalina Hamid, Wan Zurinah Wan Ngah

**Affiliations:** I Department of Biochemistry, Faculty of Medicine, Universiti Kebangsaan Malaysia, Kuala Lumpur, Malaysia.; II Sime Darby Foods & Beverages Marketing Sdn Bhd, Petaling Jaya, Selangor, Malaysia.

**Keywords:** Tocotrienol, Tocopherol, Aging, Microarray, Gene

## Abstract

**OBJECTIVES:**

This study aims to compare the differential gene expression resulting from tocotrienol-rich fraction and α-tocopherol supplementation in healthy older adults.

**METHODS:**

A total of 71 eligible subjects aged 50 to 55 years from Gombak and Kuala Lumpur, Malaysia, were divided into three groups and supplemented with placebo (n=23), α-tocopherol (n=24) or tocotrienol-rich fraction (n=24). Blood samples were collected at baseline and at 3 and 6 months of supplementation for microarray analysis.

**RESULTS:**

The number of genes altered by α-tocopherol was higher after 6 months (1,410) than after 3 months (273) of supplementation. α-Tocopherol altered the expression of more genes in males (952) than in females (731). Similarly, tocotrienol-rich fraction modulated the expression of more genes after 6 months (1,084) than after 3 months (596) and affected more genes in males (899) than in females (781). α-Tocopherol supplementation modulated pathways involving the response to stress and stimuli, the immune response, the response to hypoxia and bacteria, the metabolism of toxins and xenobiotics, mitosis, and synaptic transmission as well as activated the mitogen-activated protein kinase and complement pathways after 6 months. However, tocotrienol-rich fraction supplementation affected pathways such as the signal transduction, apoptosis, nuclear factor kappa B kinase, cascade extracellular signal-regulated kinase-1 and extracellular signal-regulated kinase-2, immune response, response to drug, cell adhesion, multicellular organismal development and G protein signaling pathways.

**CONCLUSION:**

Supplementation with either α-tocopherol or tocotrienol-rich fraction affected the immune and drug response and the cell adhesion and signal transduction pathways but modulated other pathways differently after 6 months of supplementation, with sex-specific responses.

## INTRODUCTION

Vitamin E is composed of eight naturally occurring isoforms, namely, four tocopherols (α-, β-, δ-, and γ) and four tocotrienols (α-, β-, δ-, and γ), which differ by the number and position of the methyl groups on the chromanol ring and the level of saturation in their side chains ( 1 ). The tocopherols have a saturated phytyl tail, while the tocotrienols have an unsaturated isoprenoid tail ( 2 ). In addition to its antioxidant properties, vitamin E also exerts non-antioxidant functions, such as modulating DNA repair systems, gene expression and signal transduction ( 3 ).

Most commercially available vitamin E supplements contain only α-tocopherol (α-TF). This isoform is commonly used because of its high bioavailability; compared to other isoforms, it is easily recognized by the hepatic α-TF transfer protein (TTP), and it is enriched in human plasma and tissues ( 4 ). However, α-TF alone may not be the best formulation for vitamin E supplementation, because the intake of vitamin E should reflect its natural composition, which consists of all isomers ( 5 ). Emerging evidence has shown that tocotrienol has higher antioxidant activity ( 4 ) and more potent antihypercholesterolemic ( 6 ), anti-inflammatory ( 7 ), antithrombotic ( 8 ), anticancer ( 9 , 10 , 11 ), hepatoprotective ( 12 ) and neuroprotective ( 13 ) properties than tocopherol.

A previous study by Chin et al. ( 14 ) reported that vitamin E responses were age-dependent; tocotrienol-rich fraction (TRF) supplementation resulted in a greater reduction in total DNA damage in older adults (>50 y) than in younger adults (35-49 y). In addition, improved serum lipid profiles and levels of vitamins E and C, as well as decreased levels of protein and lipid damage, were observed in older adults supplemented with TRF ( 5 ). Furthermore, Eng et al. ( 15 ) found that changes in protein expression with TRF supplementation were more profound in older individuals (49-51 y) than in younger individuals (34-36 y). According to Marino et al. ( 16 ), nutrition could influence the health of males and females differently due to multifactorial inputs, including gene repertoires, sex steroid hormones, ontogenetic developments, environmental factors and differences in the bioavailability, metabolism, distribution, and elimination of nutrients. In addition to the uncertainty surrounding the sex-specific responses to supplementation, it is still unclear whether the previously observed effects were entirely due to tocotrienol, because TRF contains traces of tocopherol. Therefore, the aim of this study was to compare the effects of α-TF with TRF supplementation in male and female subjects aged 50-55 years.

## METHODS

### Study Design

This study is a randomized, single-blinded, placebo-controlled trial approved by the Research and Ethics Committees of the Faculty of Medicine, Universiti Kebangsaan Malaysia (UKM). Volunteers who gave informed consent were screened to ensure that they met the study’s inclusion (age 50-55 years, healthy, nonsmoker, no significant clinical diseases, and no current use of medications, alcohol or supplements) and exclusion criteria. A full physical examination, previous history of medical illnesses and blood hematology profile were also obtained from the volunteers to confirm suitability. Of the 523 screened volunteers, 71 fulfilled all inclusion and exclusion criteria. The volunteers (26 males and 45 females) recruited from Gombak and Kuala Lumpur in Malaysia were distributed equally into three groups receiving either placebo (olive oil, n=23), α-TF (400 IU/day, n=24) or TRF (150 mg/day, n=24) capsules daily after dinner to ensure proper absorption. The treatment was double blinded throughout the study period until all data were collected, after which the randomization code was exposed. The subjects’ food intake frequency was assessed using a modified questionnaire by Chee et al. ( 17 ) before blood was sampled at the UKM Medical Centre. The subjects were encouraged to maintain their usual diet and lifestyle throughout the study period. Compliance was checked by counting the remaining capsules at each visit. Blood sampling was performed at baseline (month 0) and at 3 and 6 months of supplementation. Peripheral blood mononuclear cells (PBMCs) were isolated from whole blood for the evaluation of gene expression using a microarray. Of the recruited subjects, five from each group and each sex were used for the microarray analysis.

### Vitamin E Capsules for Supplementation

All commercial capsules were prepared and supplied by Sime Darby Bioganic Sdn. Bhd. (previously known as Golden Hope Bioganic), Kuala Langat, Selangor, Malaysia. The TRF (Gold TriE^®^ Tocotrienol) soft gelatin capsules consisted of approximately 74% tocotrienol and 26% tocopherol extracted from palm oil. The α-tocopherol capsules contained 100% α-tocopherol, while the placebo capsules contained only olive oil.

### Isolation of PBMCs

Briefly, a total of 35 ml whole blood was added to Lymphoprep solution (Biodiagnostic, US) and centrifuged at 1800 rpm for 30 min at room temperature (25°C). The tube was removed carefully from the centrifuge (Axis-Shield PoC, Norway), where the resulting four layers were observed: a clear supernatant top layer, an opaque fluid upper middle layer containing the PBMCs, a lower middle layer containing Lymphoprep, and a bottom layer consisting of erythrocytes and granulocytes. The layer containing the PBMCs was transferred into a new tube, washed three times with phosphate-buffered saline (PBS) and centrifuged at 1,500 rpm for 10 min at room temperature. The pellet was resuspended in 3 ml of TRI-Reagent (Invitrogen Life Technologies, Carlsbad, CA) and stored at -80°C until use.

### RNA Extraction from PBMCs and Quality Assessment

Total RNA was extracted from PBMCs and purified using an RNeasy Mini Kit (Qiagen, Valencia, CA). Extraction was conducted according to the kit manual. The RNA purity and concentration were determined by measuring the absorbance at 260 nm (A260) and 280 nm (A280) using a NanoDrop ND-1000 (Thermo Fisher Scientific, USA), while the RNA integrity was assessed by an RNA 6000 Nano LabChip Kit using an Agilent 2100 bioanalyzer (Agilent Technologies, Palo Alto, CA).

### Gene Expression Profiling (Microarray)

The microarray target sample processing, target hybridization, washing, staining, and scanning steps were performed according to the manufacturer’s protocol (Illumina Inc., San Diego, CA). Briefly, samples of 50 ng of total RNA were amplified and transcribed in vitro into biotinylated cRNA using an Epicentre TargetAmpTM-Nano Labeling Kit (Ambion, Inc., Austin, TX). The samples were then washed using an RNeasy MinElute Cleanup Kit (Qiagen, Valencia, CA). The purified cRNA was loaded into an Illumina HumanHT-12 BeadChip and hybridized overnight (17 h) in a 58°C hybridization oven (Illumina Hybridization Oven). Unhybridized and nonspecifically bound cRNAs were removed and washed using the buffer provided in the BeadChip Hybridization Kit. The specifically bound, biotinylated cRNAs were visualized by Cy3-streptavidin, and the fluorescent signals were scanned using Illumina iScan Technology. Finally, the raw data were extracted from the scanned images and analyzed with GenomeStudio, Partek and Pathway Studio 11.2 software.

### Quantitative Real-time Reverse Transcription PCR (RT-qPCR)

RT-qPCR was performed to quantitate and verify the level of mRNA expression found in the microarray experiment. The RNA samples used for microarray analysis were subjected to RT-qPCR using a One-Step RT-qPCR Kit with SYBR Green (Bio-Rad, Canada) according to the manufacturer’s protocol. The fluorescence signals were measured using an iCycler iQ5 Real-Time PCR Detection System (Bio-Rad Laboratories, USA). The primers for the selected transcripts were designed using National Center for Biotechnology Information (NCBI) (http://www.ncbi.nlm.nih.gov) resources. To maximize PCR efficiency, amplicons were designed to be fewer than 250 base pairs in length with a common melting temperature (56-61°C) for all primers. The efficiency and specificity of each primer set were confirmed using a standard curve (Ct value *versus* the serial dilution of total RNA) and agarose gel electrophoresis. The primer sequences (forward/reverse) used for RT-qPCR are shown in Table 1 . Briefly, the reaction was performed by mixing the samples with 1 µl of total RNA (100 ng), 2 µl of the primers (forward & reverse) and 17 µl of master mix (10 µl of 1×QuantiTect SYBR® Green solution, 0.2 µl QuantiTect RT Mix, and 6.8 µl RNase-free water; all provided in the kit) and incubated in the iCycler instrument with the following reaction profile: cDNA synthesis for 10 min at 50°C; predenaturation for 2 min at 95°C; and PCR amplification for 38 cycles of 30 sec at 94°C and extension for 30 sec at 61°C. Each sample was amplified in duplicate, and the results were normalized to those of GAPDH as a reference gene. The relative expression values of the selected genes were calculated using the following equation:

**Table 1:** Primer sequences for real-time quantitative RT-PCR.

Accession number^a^	Genes	Sequence (5'→3') Sense and antisense primers	Product size (base pairs, bp)
NM_002046	*GAPDH*	F: tcc ctg agc tga acg gga ag R: gga gga gtg ggt gtc gct gt	217
NM_000546	*TP53*	F: tgt gac ttg cac gta ctc cc R: acc atc gct atc tga gca gc	199
NM_006167	*NKX3-1*	F: ccc aca ctc agg tga tcg ag R: gtc tcc gtg agc ttg agg tt	103
NM_003879	*CFLAR*	F: ttg tgc cgg gat gtt gct at R: aga gca gtt cag cca agt cc	109
NM_198589	*BSG*	F: ttc atc tac gag aag cgc cg R: cag gaa gag ttc ctc tgg cg	131
NM_003862	*FGF18*	F: aag tcc gga tca agg gca ag R: tca ggg ccg tgt agt tgt tc	138
NM_002423	*MMP7*	F: gga gct cat ggg gac tcc ta R: tcc agc gtt cat cct cat cg	115
NM_001825	CKMT2	F: gtt cga cga gca tta cgt gc R: cag tga tgg cca cgt tct ct	122
NM_021116	*ADCY1*	F: ggt ttg gca gct cct ttt gg R: gga acg cct tcc tct gtg aa	245

^a^eSource of accession number is the NCBI, http://www.ncbi.nlm.nih.gov/gene/ .

*GAPDH*: glyceraldehyde-3-phosphate dehydrogenase; *TP53*: tumor protein p53; *NKX3-1*: androgen-regulated homeobox gene; *CFLAR*: apoptosis regulator; *BSG*: basigin-encoding plasma membrane protein in spermatogenesis; *FGF18*: fibroblast growth factor 18; *MMP7*: matrix metalloproteinase-7; *CKMT2*: mitochondrial creatine kinase-2; *ADCY1*: adenylyl cyclase type 1.

$$\begin{array}{c} \mathrm{Relative}\;\mathrm{expression}\;\mathrm{value}\;(\mathrm{REV})\;=\;2{\;}^{\mathrm{Ct}\;\mathrm{value}\;\mathrm{of}\;\mathrm{GAPDH}\;-\;\mathrm{Ct}\;\mathrm{value}\;\mathrm{of}\;\mathrm{selected}\;\mathrm{gene}}\;\\ \mathrm{Ct}\;=\;\mathrm{threshold}\;\mathrm{cycle}\;\\ \mathrm{Fold}\;\mathrm{change}\;(\mathrm{FC})\;=\;{\mathrm{REV}}_{\mathrm{treatment}}/{\mathrm{REV}}_{\mathrm{control}} \end{array}$$

### Statistical Analysis

The comet assay and RT-qPCR data were analyzed using Statistical Package for Social Sciences 16.0 (SPSS, Inc., Chicago, IL, USA). ANOVA was used to compare the differences between groups, with *p* <0.05 as the significance level. The data are reported as the means±SEMs. Genes that did not meet the criteria for differential expression in the microarray analysis were removed by computing a 3-way ANOVA with a significance level of *p* <0.05. Genes that changed in expression by less than 1.5-fold were also removed from subsequent analysis. Gene Set Enrichment Analysis (GSEA) was performed using a nonparametric Kolmogorov-Smirnov statistical test to calculate the *p* value of the biological processes/pathways across the whole database most affected by supplementation based on the gene regulation data in our experimental dataset. Fisher’s exact test was then conducted to determine the specific biological processes/pathways affected by supplementation according to the list of significant genes. Functional attribution was made by referring to online databases, and biological interpretation was obtained from the literature.

## RESULTS

### Subject Demographics

The 26 male and 45 female subjects recruited from the Gombak and Kuala Lumpur area were not significantly different in body mass index (BMI), blood pressure, glucose or total cholesterol throughout the study period ( Table 2 ).

**Table 2:** Demographic data of the study groups.

	Supplementation Groups		

	Placebo (Control)	α-Tocopherol (α-TF)	Tocotrienol-Rich Fraction (TRF)
Age (years)	52.2 ± 2.1	52.5 ± 2.5	53.4 ± 1.5
Sex (male/female)	8/15	9/15	9/15
Body Mass Index (BMI) [kg/m2]	26.4 ± 0.8	26.2 ± 0.8	25.3 ± 0.7
Blood Pressure (BP) [mmHg]:			
- Systolic	123.5 ± 2.5	131.1 ± 3.3	130.6 ± 2.9
- Diastolic	78.3 ± 2.1	81.3 ± 1.5	80.0 ± 2.1
Pulse (beats per minute)	73 ± 2	72 ± 2	74 ± 2
Fasting Blood Sugar (FBS)	5.09 ± 0.1	4.94 ± 0.1	4.91 ± 0.1
Total Cholesterol (Ch)	5.2 ± 0.2	5.6 ± 0.1	5.6 ± 0.1

### Modulatory effects of Vitamin E on Gene Expression and Pathways

A total of 71 individual BeadChips were analyzed using Partek software. Further analysis with a 3-way ANOVA using Partek and Pathway Studio 11.2 software revealed that at *p* <0.05, the total number of up- and downregulated genes modulated by 3 months of α-TF and TRF supplementation was similar to the number modified by 6 months of supplementation. Further analysis by sex revealed that more genes were modulated in the male subjects after 3 months than after 6 months of supplementation with either vitamin supplement. However, after filtering the gene list at a cutoff fold change of 1.5-fold, the total number of genes modulated by the vitamins was slightly lower after 3 months than after 6 months of supplementation in both male and female subjects ( Table 3 ). Considering both sexes and both supplementation time points, α-TF supplementation modulated a total of 1,683 genes; TRF, 1,680.

**Table 3:** Total number of up- and downregulated genes modulated in male and female subjects after 3 and 6 months of α-TF and TRF supplementation.

Group	α-TF *vs* Placebo				TRF *vs* Placebo				
	
	Male		Female		Male		Female		
			
	3 months	6 months	3 months	6 months	3 months	6 months	3 months		6 months
Differentially expressed genes with *p* <0.05:									
Up	1,258	935	737	966	629	647		530	681
Down	951	1,042	719	1,152	717	551		488	592
Total	2,209	1,977	1,456	2,118	1,346	1,198		1,018	1,273
Differentially expressed genes with *p* <0.05 and fold change ≥1.5:									
Up	44	338	61	270	150	247		107	277
Down	96	474	72	328	224	278		115	282
Total	140	812	133	598	374	525		222	559

**Figure 1:**
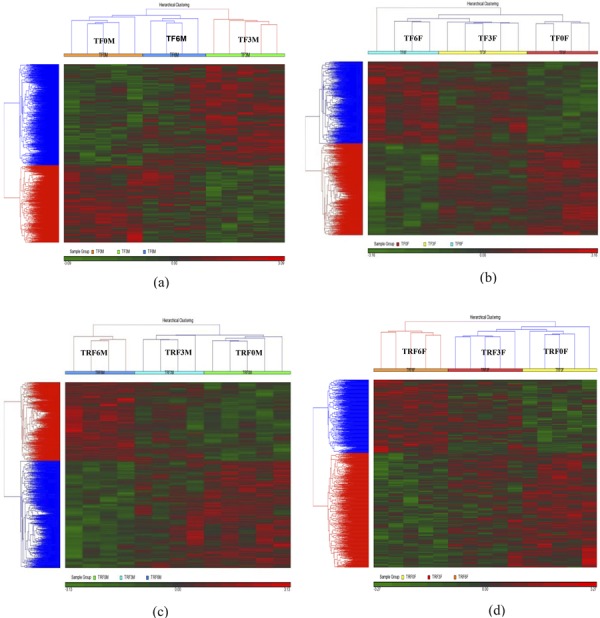
Hierarchical clustering of α-TF and TRF supplementation. Similar gene expression profiles were joined to form a group. The expression profiles of the corresponding genes were considered to be significantly different at a fold change of ≥1.5 and *p* <0.05. Red indicates overexpressed genes, while green indicates inhibited genes. (a) α-TF supplementation in males after 3 and 6 months compared to 0 months. (b) α-TF supplementation in females after 3 and 6 months compared to 0 months. (c) TRF supplementation in males after 3 and 6 months compared to 0 months. (d) TRF supplementation in females after 3 and 6 months compared to 0 months.

Hierarchical clustering showed that all samples from the same supplementation group (according to the supplement type, time point and sex) grouped well based on the similarity of the gene expression profiles ( Figure 1 ). GSEA was conducted on a list of differentially expressed genes ( *p* <0.05) with a fold change of >1.0 (obtained from Partek analysis) to identify the functional categories of the genes. Fisher’s exact test was performed to compute the *p* values in order to determine the overlap between the entities (gene set) and pathways. The gene ontology (pathway) was ranked based on the highest *p* value (Supplementary Table S1-S8).

**Figure 2:**
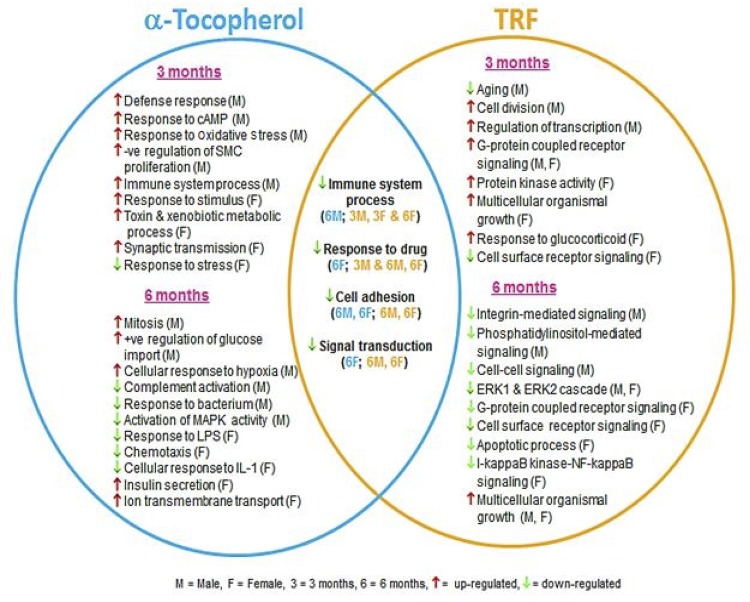
Biological processes significantly modulated in both male and female subjects after 3 and 6 months of α-TF and TRF supplementation.

Three months of supplementation with α-TF upregulated the immune system, responses to cyclic adenosine monophosphate (cAMP) and oxidative stress pathways as well as the negative regulation of smooth muscle cell proliferation pathway in the male subjects ( Figure 2 ; Supplementary Table S1). Six months of supplementation with α-TF upregulated the mitosis, glucose import and cellular response to hypoxia pathways but downregulated the responses to bacterium, complement activation and mitogen-activated protein kinase (MAPK) activity pathways in the male subjects ( Figure 2 ; Supplementary Table S2). In the female subjects, α-TF supplementation upregulated the toxin metabolic processes, responses to stimuli, xenobiotic metabolic processes and synaptic transmission pathways but downregulated the cellular responses to stress pathway after 3 months ( Figure 2 ; Supplementary Table S3). Supplementation with α-TF for 6 months upregulated the insulin secretion and transmembrane ion transport pathways but downregulated the responses to lipopolysaccharide (LPS), chemotaxis, and interleukin-1 (IL-1) pathways in the female subjects ( Figure 2 ; Supplementary Table S4).

For TRF, 3 months of supplementation in the male subjects upregulated the cell division, regulation of cell transcription and G protein-coupled receptor signaling pathways ( Figure 2 ; Supplementary Table S5). However, 6 months of supplementation with TRF upregulated only the growth pathway but downregulated the integrin-mediated signaling, phosphatidylinositol-mediated signaling, cell-cell signaling and extracellular signal-regulated kinase 1/2 (ERK1/2) cascade pathways in the male subjects ( Figure 2 ; Supplementary Table S6). Among the female subjects, 3 months of supplementation with TRF upregulated the G protein-coupled receptor signaling, protein kinase activity, and growth and responses to glucocorticoid pathways but downregulated the cell surface receptor signaling pathway ( Figure 2 ; Supplementary Table S7). Six months of TRF supplementation upregulated only the growth pathway in the female subjects but downregulated the ERK1/2 cascade, G protein-coupled receptor signaling, cell surface receptor signaling, apoptosis and I-kappa B kinase-nuclear factor-kappa B signaling pathways ( Figure 2 ; Supplementary Table S8).

The biological processes that were modulated similarly by α-TF and TRF supplementation were the immune system, drug response, cell adhesion and signal transduction processes. These processes were downregulated in both males and females mainly after 6 months of supplementation.

### Gene Validation

**Figure 3:**
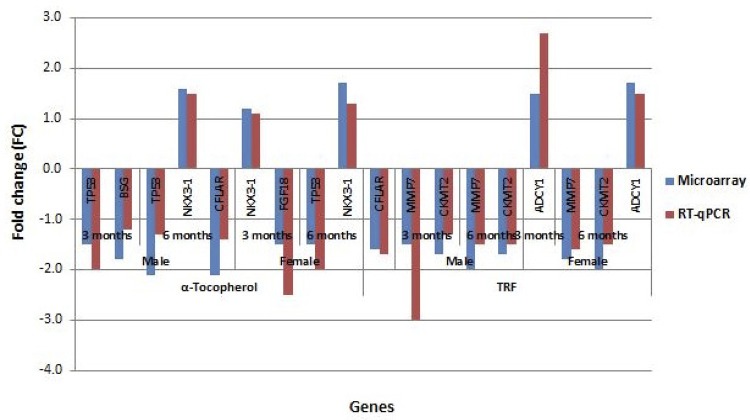
Comparison of gene expression between the microarray and RT-qPCR results.

To validate the microarray results, the mRNA transcript levels of six downregulated genes and one upregulated gene were quantified by real-time RT-qPCR using the PBMC samples from each subject. Genes were selected based on their function and their identification as a major and significantly differentially regulated gene in any biological process generated by GSEA and Fisher’s exact test. Overall, the fold changes in the differentially expressed genes in the RT-qPCR analysis were consistent and in agreement with the microarray analysis results ( Figure 3 ).

## DISCUSSION

In the elderly, the intake of essential macro- and micronutrients from the diet is usually inadequate. The deficiency of essential nutrients in aging is related to the global impairment of immune functions, metabolic harmony and antioxidant defense ( 18 ). With increasing age, the production of reactive oxygen species (ROS) is also increased due to an imbalance between antioxidant defense and ROS production. The resulting oxidative stress damages biomolecules such as DNA, protein and lipids, which eventually contributes to age-related diseases ( 19 ). Thus, supplementation with micronutrients with antioxidant properties, such as vitamin E, could prevent oxidative stress and molecular injury in aging.

The effects of α-TF and TRF on healthy older adults were compared in this study, and our results showed that each vitamin modulated the expression of different genes and regulated different pathways. Although the TRF supplements used in this study also contained α-tocopherol, the responses were different from the responses to α-TF supplementation alone. In the male subjects, α-TF supplementation for 3 months significantly stimulated the defense response pathway ( *p* <0.007). Although this pathway was stimulated, the expression of most related genes (FC>1.5) decreased. The expression of the *CD3* ε *,* or CD3-epsilon, gene (Entrez ID: NM_000733) (http://www.ncbi.nlm.nih.gov/gene/) was found to decrease with the highest fold change (1.6-fold). The CD complex plays an important role in coupling antigen recognition to several intracellular signal transduction pathways, such as T cell receptor (TCR) signal transduction ( 20 ). According to Li et al. ( 21 ), increased *CD3* ε expression is related to the severity of aplastic anemia, which is an autoimmune disease. The results of this study suggest that α-TF induces a cellular defense response against pathogenic conditions by decreasing the expression of *CD3* ε *,* which plays a role in autoimmune diseases. In addition, α-TF exerts an antiproliferative effect by upregulating the response to cAMP ( 11 ) and downregulating smooth muscle cell proliferation ( 22 ). The role of this vitamin as an antioxidant is also established, as the oxidative stress pathway was upregulated ( 23 ). The most significant pathway modulated after 6 months of α-TF supplementation was the mitosis pathway ( *p* <0.008), which plays a crucial role in the cell cycle ( 24 ). Upregulated mitosis shows that α-TF promotes cell cycle progression and cell division. The expression of the gene *SNX9* , or sorting nexin 9 (Entrez ID: NM_016224), was found to increase with the highest fold change (2.1-fold) in this pathway. According to Ma and Chircop ( 25 ), the SNX9 protein is essential for the progression and completion of mitosis, and the depletion of this protein induces multinucleation (an indication of cytokinesis failure) and the accumulation of cytokinetic cells. In addition, α-TF may have anti-inflammatory effects, as supplementation was found to downregulate the cell adhesion and cell response to bacterium pathways and inhibit proliferation by decreasing the activation of the MAPK activity pathway ( 26 ) in healthy male subjects.

In female subjects, the most significant pathway modulated by α-TF after 3 months of supplementation was the toxin metabolic process pathway ( *p* <0.008). The upregulation of this pathway shows that α-TF may promote detoxification by eliminating various types of toxins, such as mutagens, carcinogens, drugs, excessive hormones and chemicals ( 27 ), from the body after as few as 3 months of supplementation. At this time point, α-TF was also found to upregulate xenobiotic metabolism, which is also related to the detoxification mechanism. Although both pathways were upregulated, the expression of most of the genes involved in these pathways was found to decrease. The expression of the *CYP1A1* , or cytochrome P450 family 1 subfamily A member 1, gene (Entrez ID: NM_000499) was found to decrease with the highest fold change (1.5-fold). This gene encodes the P450-1A1 (CYP1A1) enzyme, which has aryl hydrocarbon hydroxylase activity ( 28 ). This enzyme converts polycyclic aromatic hydrocarbons (PAHs) to aryl epoxide carcinogens ( 29 ) and participates in estrogen metabolism by catalyzing the 2-hydroxylation of estradiol, which results in free radical and DNA adduct production ( 30 ). The decreased expression of this gene may inhibit the production of ROS that lead to cancer development. After 6 months of supplementation, α-TF significantly downregulated the signal transduction pathway ( *p* <0.007). Signal transduction (also known as cell signaling) is a process of (chemical or physical) signal transmission through a cell that results in a response that may alter cell metabolism or gene expression ( 31 ). In this pathway, the expression of the *TP53* , or tumor protein 53, gene (Entrez ID: NM_000546) was downregulated by 1.5-fold. This gene encodes a tumor suppressor protein that has a DNA binding site and transcriptional activation and oligomerization domains that respond to diverse cellular stresses to regulate targeted genes by inducing apoptosis, cell cycle arrest, DNA repair, senescence, or metabolic changes. Ishak et al. ( 32 ) reported that the increased expression of *TP53* was detected in 50% of gallbladder carcinomas. According to Barabutis et al. ( 33 ), p53, which functions as a tumor suppressor, promotes apoptosis, cell cycle arrest and senescence under stress conditions. In this study, the decreased expression of the *TP53* gene may be related to a decreased stress response with α-TF supplementation, leading to a downregulated signaling cascade as well as a decreased response to LPS, IL-1 and chemotaxis. α-TF may also exert anti-inflammatory effects by downregulating the cell adhesion ( 34 ) and cellular responses to iIL-1 pathways in healthy female subjects.

The G protein-coupled receptor signaling pathway was upregulated after 3 months of TRF supplementation in male subjects (0.007). G protein-coupled receptors (GPCRs) are located at the cell surface to convert endogenous signals or stimuli into a series of cellular responses ( 35 ). Although this pathway was upregulated by supplementation, most of the significant genes (FC>1.5) involved in this pathway were found to be downregulated, especially the *GPR110* , or the adhesion G protein-coupled receptor F1, gene (Entrez ID: NM_025048), which was downregulated with the highest fold-change (2.4-fold). *GPR110* is an orphan GPCR that has been identified as an oncogene overexpressed in some lung and prostate cancers and is used as a disease marker and therapeutic target for both types of tumors ( 36 ). Short-term supplementation with TRF may also promote the cell cycle by upregulating the cell division pathway and delay aging by downregulating the aging pathway. The most significant pathway upregulated after 6 months of TRF supplementation was the multicellular organismal development pathway ( *p* =0.006). The *CTNDD2* , or delta 2 catenin, gene (Entrez ID: NM_001332) was upregulated with the highest fold change (1.9-fold). This gene encodes a δ-catenin and is involved in the regulation of dendrite function and neuronal migration in the mature cortex ( 37 ). Intragenic *CTNND2* deletion is found in patients with isolated intellectual disability ( 38 ). Based on these results, TRF has been suggested to have a neuroprotective effect in male subjects by downregulating the ERK1/2 cascade ( 13 ). TRF also exerts anti-inflammatory effects by decreasing cell adhesion ( 39 ) and suppressing integrin-mediated signaling, phosphatidylinositol-mediated signaling and cell-cell signaling pathways in male subjects.

In the female subjects, the biological process most significantly modulated after 3 months of TRF supplementation was the activation of protein kinase pathway ( *p* <0.008). Protein kinase activity is modified by other proteins via phosphorylation, which results in the alteration of protein function ( 40 ). Though this process was stimulated by TRF supplementation, the expression of all the significant genes (FC>1.5) involved was decreased. The expression of the epidermal growth factor (EGF) gene was found to decrease with the highest fold change (2.2-fold) after supplementation. Decreased EGF expression is beneficial for the prevention of breast cancer because EGF is a potent mitogen for normal and neoplastic mammary epithelial cells ( 12 ). Indeed, McIntyre et al. ( 41 ) reported that tocotrienols specifically inhibit EGF-dependent mitogenesis in preneoplastic and neoplastic mammary epithelial cells. Like α-TF supplementation, TRF supplementation for 6 months downregulated the signal transduction pathway significantly ( *p* <0.006). Among the genes in this pathway, TRF downregulated the *LTB*_4_ *R2* , or leukotriene B4 receptor 2, gene (Entrez ID: NM_001164692) with the highest fold change (2.4-fold). LTB_4_ is reported to be a potent proinflammatory lipid mediator that is overproduced in the pathogenesis of several inflammatory diseases ( 42 ), such as rheumatoid arthritis, bronchial asthma, ischemic renal failure, psoriasis and inflammatory bowel diseases ( 43 ). Studies have shown that LTB_4_ and its receptors critically regulate tumor progression by promoting cell proliferation, migration, survival and metastasis ( 44 , 45 ). Kim et al. ( 46 ) reported that the expression of the *LTB*_4_ *R*_2_ gene (also known as *BLT2* ) was upregulated in MCF-7 (a human breast cancer cell line)/DOX (doxorubicin) cells, whereas cotreatment with a *BLT2* inhibitor markedly reduced tumor growth in an *in vivo* MCF-7/DOX model. TRF also exerts antiapoptotic effects by downregulating the apoptotic process pathway ( 47 ); furthermore, it shows neuroprotective properties by decreasing the ERK1/2 cascades ( 15 , 16 ) and anti-inflammatory and anticancer properties by downregulating the I-kappa B kinase-NF-kappa B signaling ( 48 ) and cell adhesion pathways ( 39 ) in healthy female subjects.

Overall, supplementation with either α-TF or TRF modulated the immune system, response to drug, cell adhesion and signal transduction pathways. de Magalhaes et al. ( 49 ) reported that age-related gene changes most notably involve an overexpression of immune response genes. Theriault et al. ( 39 ) reported that the anti-inflammatory and cardioprotective effects of tocotrienols are mediated through the ability of tocotrienols to downregulate the expression of adhesion molecules. For example, α-TF has been reported to prevent inflammation and atherosclerosis by reducing monocyte cell adhesion activity ( 50 ). Zingg ( 3 ) reported that vitamin E specifically modulates signal transduction in order to scavenge free radicals by directly interacting with signal transduction enzymes or by reducing ROS- and reactive nitrogen species (RNS)-induced damage to enzymes. Furthermore, Khanna et al. ( 51 ) showed that the neuroprotective effect of α-tocotrienol did not result from its antioxidant activity but from the suppression of specific signal transduction mediators. In this study, the ability of both types of vitamin E to suppress the signal transduction pathway may have led to the downregulation of most biological processes, especially in female subjects after 6 months of supplementation. Although the distribution of males and females in each supplementation group was similar, the study was limited because the number of females was greater than the number of males. Thus, further research encompassing a larger sample size with an equal distribution of males and females is necessary to confirm the sex-specific effects of α-TF and TRF supplementation observed in this study.

Both α-TF and TRF supplementation had similar effects on the immune system, drug response, cell adhesion and signal transduction pathways. However, TRF supplementation showed a more pronounced effect than α-TF in modulating the expression of genes in signaling pathways. The antioxidative and anti-inflammatory properties of TRF observed in female subjects may be attributed to the downregulation of the apoptotic pathway, ERK1/2 cascades and NF-kB pathway after 6 months of supplementation.

## Appendix

**Supplementary Table 1:** List of biological processes and genes significantly modulated in male subjects after 3 months of α-TF supplementation

BIOLOGICAL PROCESS/ PATHWAY	GENE SET ENRICHMENT ANALYSIS (GSEA)		FISHER’S EXACT TEST: ENRICHED PATHWAYS		
	Normalized enrichment score (NES)	*p* -value	Overlapping genes in database	Total entities	*p* -value
Defense response	1.54 (↑)	0.007	*MLF2* (↓) *;CEBPE* (↓) *;LSP1* (↓) *;DARC* (↓) *;CLEC1A* (↓) *; CD3E* (↓) *;TNFRSF4* (↑) *;ITGAM* (↓) *;MYC* (↑) *;ELAVL1* (↑)	98	0.02
Response to cAMP	1.50 (↑)	0.008	*CDK2* (↑) *;PEBP1* (↑) *;BIRC2* (↓) *;CITED1* (↓) *;BSG* (↓) *; PTK2B* (↓) *;CD4* (↓)	67	0.03
Response to oxidative stress (OS)	1.40 (↑)	0.009	*PARK7* (↑) *;PPID* (↑) *;TP53* (↓) *;JAK2* (↓) *;PEBP1* (↑) *; PXDN* (↓) *;SIRT1* (↓) *;COQ7* (↑) *;ALAD* (↑) *;ERCC3* (↑) *; GPX4* (↓)	138	0.002
-ve regulation of smooth muscle cell proliferation	1.65 (↑)	0.01	*FGFR2* (↓) *;CALCRL* (↓) *;NOX1* (↑) *;FGF2* (↓)	62	0.03
+ve regulation of reactive oxygen species metabolic process	1.63 (↑)	0.01	*ROMO1* (↓) *;TP53* (↓) *;PID1* (↓) *;PTK2B* (↓)	31	0.04
Immune system process	1.33 (↑)	0.01	*CD4* (↓) *;SEMA4A* (↓) *;JAK2* (↓) *;INPP5D* (↑) *;ITK* (↑) *; CFP* (↓) *;ORAI1* (↓) *;IRF1* (↑)	365	0.001

**Supplementary Table 2:** List of biological processes significantly modulated in male subjects after 6 months of α-TF supplementation

BIOLOGICAL PROCESS/ PATHWAY	GENE SET ENRICHMENT ANALYSIS (GSEA)		FISHER’S EXACT TEST: ENRICHED PATHWAYS		
	Normalized enrichment score (NES)	*p* -value	Overlapping genes in database	Total entities	*p* -value
Mitosis	1.32 (↑)	0.008	*KLHL9* (↓) *;NEK2* (↓) *;TPX2* (↓) *;KIF11* (↓) *;KIF18B* (↑) *; SNX9* (↑) *;CDC25A* (↓) *;VCPIP* (↓) *;CDKN1A* (↑)	252	0.009
Positive regulation of glucose import	1.67 (↑)	0.009	*IRS1* (↑) *;IRS2* (↑) *;GLP1R* (↑)	35	0.03
Response to bacterium	-1.53 (↓)	0.009	*NLRP6* (↓) *;IL6* (↑)	37	0.04
-ve regulation of immune response^†TRF3M, TRF3F & TRF6F^	1.64 (↑)	0.01	*EXO1* (↓) *;INPP5D* (↑) *;PDCD1* (↓) *;CD180* (↓) *;MR1* (↑) *; TNFSF18* (↓)	14	0.002
Complement activation	-1.51 (↓)	0.01	*MBL2* (↑) *;C8B* (↑) *;CFLAR* (↓)	28	0.02
Activation of MAPK activity	-1.51 (↓)	0.01	*PTPRC* (↓) *;AVPI1* (↑) *;HBEGF* (↑)	99	0.01
Cell adhesion^†TF6F, TRF6M, TRF6F^	-1.22 (↓)	0.01	*PCDH19* (↓) *;PKN2* (↓) *;DCBLD2* (↓) *;COL15A1* (↓) *; CDH15* (↑) *;CLDN1* (↑) *;COL8A1* (↑) *; THBS4* (↑) *;CUZD1* (↓) *;RHOB* (↑) *; CNTN2* (↑) *; RGMB* (↑) *;DH9* (↓) *;CDH12* (↑) *;SDK2* (↓)	593	0.001
Cellular response to hypoxia	1.28 (↑)	0.01	*NKX3-1* (↑) *;TP53* (↓) *;ITPR2* (↓) *;VEGFA* (↑) *;SIRT1* (↓)	99	0.06

†Similar effects to TRF 3 months male (TRF3M), TRF 3 months female (TRF3F), TRF 6 months female (TRF6F), α-TF 6 months female (TF6F), and TRF 6 months male (TRF6M)

**Supplementary Table 3:** List of biological processes significantly modulated in female subjects after 3 months of α-TF supplementation

BIOLOGICAL PROCESS/ PATHWAY	GENE SET ENRICHMENT ANALYSIS (GSEA)		FISHER’S EXACT TEST: ENRICHED PATHWAYS		
	Normalized enrichment score (NES)	*p* -value	Overlapping genes in database	Total entities	*p* -value
Toxin metabolic process	1.61 (↑)	0.008	*FMO2* (↓) *;CYP1A1* (↓)	9	0.02
Cellular response to stress	-1.62 (↓)	0.009	*PDCD6* (↑) *;SRPX* (↑)	13	0.02
Response to stimulus	1.33 (↑)	0.01	*GUCA1A* (↑) *;MYO3A* (↑) *;RP1* (↓) *;NRL* (↓) *;LCN1* (↓) *; TULP1* (↓) *;PTN* (↓) *;KAT5* (↑)	393	0.001
Xenobiotic metabolic process	1.31 (↑)	0.01	*CYP19A1* (↓) *;FMO2* (↓) *;SULT4A1* (↓) *;ADH1C* (↓) *; CYP1A1* (↓) *;CYP2F1* (↓) *;GSTP1* (↓) *;CNGB3* (↓)	159	0.007
Positive regulation of uterine smooth muscle contraction	1.56 (↑)	0.01	*ADRA2B* (↓) *;TACR1* (↓) *;TACR2* (↓) *;IFNB1* (↓)	7	0.03
Synaptic transmission	1.20 (↑)	0.01	*GABARAP* (↓) *;KCNJ6* (↑) *;GNGT1* (↓) *;CACNG3* (↑) *; KCNC3* (↑) *;GRIA1* (↓) *; GRIN1* (↓) *;MT3* (↑)	442	0.001

**Supplementary Table 4:** List of biological processes significantly modulated in female subjects after 6 months of α-TF supplementation

BIOLOGICAL PROCESS/ PATHWAY	GENE SET ENRICHMENT ANALYSIS (GSEA)		FISHER’S EXACT TEST: ENRICHED PATHWAYS		
	Normalized enrichment score (NES)	*p* -value	Overlapping genes in database	Total entities	*p* -value
Signal transduction^†TRF6M, TRF6F^	-1.20 (↓)	0.007	*GPR151* (↑) *;AHRR* (↑) *;GPR139* (↓) *;ARR3* (↑) *;IRS2* (↑) *;OSTF1* (↓) *;HPGDS* (↑) *;RASD1* (↑) *;TAS2R7* (↓) *;IL21* (↑) *; PTCH1* (↓) *;GPR39* (↑) *;PDE6B* (↓) *;HTR2B* (↑) *;HBEGF* (↑) *;IRS1* (↑) *;GPR45* (↑) *;OR4C13* (↓) *; PKN2* (↓) *;CGA* (↑) *;ADRBK2* (↓) *;CHRNA6* (↓)	1036	0.001
Cell adhesion^†TF6M, TRF6M, TRF6F^	-1.27 (↓)	0.008	*HES1* (↑) *;PKN2* (↓) *;PCDHA4* (↓) *;EPHB4* (↑) *;HAPLN3* (↑) *; RHOB* (↑) *;CX3CL1* (↑) *;CDH9* (↓) *;RADIL* (↓) *;PCDHGB3* (↓) *; SDK2* (↓) *;PPARGC1A* (↓)	593	0.001
Response to drug ^†TRF3M, TRF6M, TRF6F^	-1.29 (↓)	0.008	*HTR2B* (↑) *;TP53* (↓) *;CYP11A1* (↓) *;JUN* (↑) *; PTCH1* (↓) *;FOS* (↑) *; CDKN1A* (↑) *;FECH* (↑) *;SOCS3* (↑) *;1L6* (↑)	450	0.01
Response to lipopolysaccharide	-1.32 (↓)	0.008	*CASP8* (↓) *;CFLAR* (↓)	208	0.009
Chemotaxis	-1.52 (↓)	0.009	*CCL16* (↓) *;HRG* (↓)	140	0.008
Ion transmembrane transport	1.40 (↑)	0.01	*CHRNA4* (↓) *;SLCO1B3* (↓) *;CHRNA6* (↓) *;SLCO6A1* (↓)	252	0.009
Insulin secretion	1.48 (↑)	0.01	*VGF* (↓) *;IRS1* (↑) *;CPLX1* (↓)	36	0.04
Cellular response to interleukin-1	-1.63 (↓)	0.01	*NKX3-1* (↑) *;RNASE7* (↓)	47	0.04

†Similar effects to TRF 6 months male (TRF6M), TRF 6 months female (TRF6F), α-TF 6 months male (TF6M), TRF 6 months male (TRF6M), TRF 6 months female(TRF6F), and TRF 3 months male (TRF3M)

**Supplementary Table 5:** List of biological processes significantly modulated in male subjects after 3 months of TRF supplementation

BIOLOGICAL PROCESS/ PATHWAY	GENE SET ENRICHMENT ANALYSIS (GSEA)		FISHER’S EXACT TEST: ENRICHED PATHWAYS		
	Normalized enrichment score (NES)	*p* -value	Overlapping genes in database	Total entities	*p* -value
G-protein coupled receptor signaling pathway^†TRF3F^	1.25 (↑)	0.007	*GPR110* (↓) *;PPARD* (↑) *;OR2D2* (↓) *;BAI1* (↑) *;RORB* (↓) *; GNA14* (↑) *;AVPR1A* (↓) *;LTB4R2* (↓)	816	0.009
Response to drug^†TF6F,TRF6M,TRF6F^	-1.26 (↓)	0.007	*GATA4* (↓) *;CHRNA3* (↓) *;MMP7* (↓) *;ADAM17* (↓) *;PAM* (↓) *; ABCA3* (↓) *;LTC4S* (↓) *;CROT* (↓) *;SOCS3* (↑) *;ABAT* (↓) *; NGF* (↓) *;MET* (↑) *;CKMT2* (↓)	450	0.02
Immune system process^†TF6M,TRF3F,TRF6F^	-1.37 (↓)	0.008	*CD300LD* (↓) *;IRAK4* (↓) *;IRGM* (↑) *;IL31RA* (↓) *;CADM1* (↓) *; KLRG1* (↓)	265	0.007
Cell division	1.37 (↑)	0.01	*MIS12* (↓) *;VPS4B* (↓) *;TACC1* (↓) *;KIF2B* (↓) *;NEDD9* (↓) *;KIF11* (↓) *; DSN1* (↓) *; SMC2* (↓) *;AURKA* (↓) *;DCLRE1A* (↓) *;FIGN* (↑) *; PARD6A* (↑) *;NUF2* (↓) *;CDC27* (↓) *;TIPIN* (↓) *;TTK* (↓)	302	0.03
Aging	-1.39 (↓)	0.01	*NGF* (↓) *;PHOX2A* (↑) *;CACNA1D* (↓)	196	0.005
Regulation of transcription	1.20 (↑)	0.01	*CITED4* (↑) *;ZNF221* (↓) *;PAX4* (↓) *;IGSF1* (↓) *;HOXD13* (↑) *; PPARD* (↑) *;HOXC12* (↓) *;CTNND2* (↑)	1092	0.02

†Similar effects to TRF 3 months Female (TRF3F), α-TF 6 months Female (TF6F), TRF 6 months Male (TRF6M), TRF 6 months Female (TRF6F), α-TF 6 months Male (TF6M), TRF 3 months Female (TRF3F)

**Supplementary Table 6:** List of biological processes significantly modulated in male subjects after 6 months of TRF supplementation

BIOLOGICAL PROCESS/ PATHWAY	GENE SET ENRICHMENT ANALYSIS (GSEA)		FISHER’S EXACT TEST: ENRICHED PATHWAYS		
	Normalized enrichment score (NES)	*p* -value	Overlapping genes in database	Total entities	*p* -value
Multicellular organismal development^†TRF6F^	1.30 (↑)	0.006	*BMP3* (↓) *;RORB* (↓) *;CDX1* (↑) *;CTNND2* (↑) *;HOXB7* (↓) *; WNT11* (↑) *;NAV1* (↑) *; INSC* (↑) *;HOXC13* (↑) *; NGEF* (↑) *;GPSM1* (↑) *;HEG1* (↓) *;KIF26B* (↓) *; CSRP3* (↑) *;PKP1* (↓) *;DCLK1* (↑)	1067	0.007
Response to drug^†TF6F,TRF3M,TRF6F^	-1.39 (↓)	0.006	*CHRNA3* (↓) *;MMP7* (↓) *;ADAM17* (↓) *;KCNJ11* (↑) *;CROT* (↓) *; CKMT2* (↓)	99	0.01
Integrin-mediated signaling pathway	-1.44 (↓)	0.007	*ADAM7* (↓) *;DOCK1* (↓)	263	0.008
Phosphatidylinositol-mediated signaling	-1.43 (↓)	0.008	*IRAK4* (↓) *;PIK3CG* (↓)	133	0.003
Cell-cell signaling	-1.40 (↓)	0.008	*BMP3* (↓) *;TSHB* (↓) *;CCL4* (↓)	263	0.006
Positive regulation of ERK1 and ERK2 cascade^†TRF6F^	-1.61 (↓)	0.009	*FGF19* (↓) *;GPR183* (↑)	101	0.005
Signal transduction^†TF6F,TRF6F^	-1.24 (↓)	0.01	*GPR151* (↑) *;PLAU* (↑) *;WNT11* (↑) *;BRS3* (↑) *;PKN3* (↓) *;AVPR1A* (↓) *; LTB4R2* (↓) *;LPAR2* (↑) *; HTR1F* (↓) *;RPS6KA2* (↓) *; KCNIP2* (↑) *;IL21* (↑) *;SPOCK3* (↓) *;BDKRB1* (↓) *;IGSF1* (↓) *; CTNND2* (↑) *;CABP4* (↓) *;GPR110* (↓) *;FSHB* (↑) *;PKP1* (↓)	1156	0.01
Cell adhesion^†TF6M,TF6F,TRF6F^	-1.37 (↓)	0.01	*CRNN* (↑) *;PKP1* (↓) *;COL19A1* (↓) *;NCAM2* (↓)	99	0.008

†Similar effects to TRF 6 months female (TRF6F), α-TF 6 months female (TF6F), TRF 3 months male (TRF3M), and α-TF 6 months male (TF6M)

**Supplementary Table 7:** List of biological processes significantly modulated in female subjects after 3 months of TRF supplementation

BIOLOGICAL PROCESS/ PATHWAY	GENE SET ENRICHMENT ANALYSIS (GSEA)		FISHER’S EXACT TEST: ENRICHED PATHWAYS		
	Normalized enrichment score (NES)	*p* -value	Overlapping genes in database	Total entities	*p* -value
Activation of protein kinase activity	1.76 (↑)	0.008	*TGFB2* (↓) *;INSR* (↓) *;TOM1L1* (↓) *;EGF* (↓)	27	0.04
Response to glucocorticoid	1.33 (↑)	0.009	*A2M* (↑) *;MSTN* (↓) *;CCKAR* (↓) *;SCGB1A1* (↑) *;INSR* (↓)	131	0.009
Cell surface receptor signaling pathway	-1.28 (↓)	0.01	*AGT* (↑) *;OSTN* (↑) *;IFNB1* (↑) *;MCHR1* (↑) *;NPPB* (↑)	240	0.007
Immune response^†TF6M,TRF3M,TRF6F^	-1.44 (↓)	0.01	*IFNB1* (↑) *;KIR2DL2* (↑) *;MADCAM1* (↑)	43	0.001
Multicellular organism growth^†TRF6M,TRF6F^	1.20 (↑)	0.01	*EFNB2* (↓) *;CDX1* (↓) *;SEMA5A* (↓) *;PPP1R9B* (↑) *;MDFI* (↑) *; SNAI2* (↓) *;FGF2* (↓) *;HOXA3* (↑) *;NHLH2* (↑) *;NPHS1* (↑) *; GSX1* (↓) *;BOLL* (↑) *;NEUROD1* (↑) *;FLG* (↑) *;WNT4* (↓)	1067	0.003
G-protein coupled receptor signaling pathway^†TRF6M^	1.45 (↑)	0.01	*SSTR4* (↑) *;AGT* (↑) *;MCHR1* (↑) *;GPR111* (↑) *;INSR* (↓) *; GAST* (↓) *;S1PR4* (↑) *; DRD5* (↑) *; NPBWR2* (↑) *; GPR132* (↑) *;CCKAR* (↓) *; HTR1E* (↓) *;RAPGEF4* (↓) *;FGF2* (↓) *;ADCY1* (↑)	824	0.01

†Similar effects to α-TF 6 months male (TF6M), TRF 3 months male (TRF3M), TRF 6 months female (TRF6F), and TRF 6 months male (TRF6M)

**Supplementary Table 8:** List of biological processes significantly modulated in female subjects after 6 months of TRF supplementation

BIOLOGICAL PROCESS/ PATHWAY	GENE SET ENRICHMENT ANALYSIS (GSEA)		FISHER EXACT TEST: ENRICHED PATHWAYS		
	Normalized enrichment score (NES)	*p* -value	Overlapping genes in database	Total entities	*p* -value
Signal transduction^†TF6F,TRF6M^	-1.20 (↓)	0.006	*DGKG* (↓) *;GPR151* (↑) *;PTGDR* (↓) *;CHRNA3* (↓) *;DOCK1* (↓) *;IGFBP4* (↑) *; RAP1B* (↓) *;CCL24* (↓) *;NDFIP2* (↓) *;LTB4R2* (↓) *; HTR1F* (↓) *;SIT1* (↑) *;RPS6KA2* (↓) *;IQGAP2* (↓) *;LGR5* (↓) *; MRGPRE* (↓) *;EPS8* (↓) *;IGSF1* (↓) *;TRAF4* (↑) *;S1PR4* (↑) *; STAT5B* (↑) *;TGFBR1* (↓) *;LTA* (↑) *;TLE2* (↑) *;GNG12* (↓)	1236	0.01
+ve regulation of apoptotic process	-1.32 (↓)	0.007	*RPS6KA2* (↓) *;TFAP4* (↑) *;ANKRD1* (↑) *;EIF5A* (↑) *;DNM1L* (↓)	344	0.008
+ve regulation of I-kappaB kinase-NF-kappaB signaling	-1.61 (↓)	0.008	*NDFIP2* (↓) *;S100B* (↓) *;PIM2* (↑)	73	0.009
+ve regulation of ERK1 and ERK2 cascade^†TRF6M^	-1.50 (↓)	0.009	*FGF19* (↓) *;DOCK1* (↓)	101	0.005
Immune response^†TF6M,TRF3M,TRF3F^	-1.50 (↓)	0.01	*CCL7* (↓) *;CCL24* (↓) *;YES1* (↓) *;SIRPG* (↑) *;CXCL9* (↓) *;C8B* (↓)	113	0.001
Response to drug^†TF6F,TRF3M,TRF6M^	-1.20 (↓)	0.01	*LYST* (↓) *;PPARGC1A* (↓) *;MMP7* (↓) *;PCK1* (↓) *;AMH* (↓) *; HSD3B2* (↑) *;CKMT2* (↓)	35	0.01
Cell adhesion^†TF6M,TF6F,TRF6M^	-1.25 (↓)	0.01	*COL12A1* (↓) *;NCAM2* (↓) *;GPNMB* (↑) *;BTBD9* (↓) *; SSX2IP* (↓) *;MMP13* (↑)	593	0.001
Multicellular organism growth^†TRF3F,TRF6M^	1.41 (↑)	0.01	*KAT2A* (↑) *;HEG1* (↓)	76	0.004
G-protein coupled receptor signaling pathway	-1.41 (↓)	0.01	*KCNJ11* (↑) *;ADCY1* (↑) *;GRIK1* (↑) *; HTR1F* (↑) *;ADCY7* (↑)	41	0.01

†Similar effects to α-TF 6 months female (TF6F), TRF 6 months male (TRF6M), α-TF 6 months male (TF6M), TRF 3 months male (TRF3M), and TRF 3 months female (TRF3F)
